# *Pseudomonas aeruginosa*: a model system for studying the behavior of unbiased flagellar motors

**DOI:** 10.1128/jb.00254-26

**Published:** 2026-08-12

**Authors:** Zhengyu Wu, Maojin Tian, Sanyuan Fu, Rongjing Zhang, Junhua Yuan

**Affiliations:** 1 Research Center of Translational Medicine, Jinan Central Hospital Affiliated to Shandong First Medical University 425700, Jinan, Shandong, China; 2 Department of Critical Care Medicine, Zibo Central Hospital Affiliated to Shandong Medical And Pharmaceutical University 117906 , Zibo, Shandong, China; 3 Hefei National Research Center for Physical Sciences at the Microscale and Department of Physics, University of Science and Technology of China 12652, Hefei, Anhui, China; Dartmouth College Geisel School of Medicine, Hanover, New Hampshire, USA

**Keywords:** *Pseudomonas aeruginosa* chemotaxis, dual-stator system, unbiased motor switching, bacterial flagellar motor

## Abstract

The flagellar motor of *Pseudomonas aeruginosa* operates in a near-unbiased manner, extending the classical paradigm established by *Escherichia coli*. Rather than altering rotational bias, *P. aeruginosa* achieves chemotaxis by symmetrically modulating the dwell times of the two rotational states. This strategy is associated with a mechanically adaptive dual-stator architecture (MotAB and MotCD) and is further modulated by c-di-GMP signaling and auxiliary proteins such as FliL and MotY. At the molecular level, the distinctive reversal behavior is thought to arise from the unique response of the switch complex (located on the rotor) to phosphorylated CheY, which alters switching kinetics rather than rotational bias. These features collectively give rise to distinct swimming behaviors, including a “wrap” state that facilitates three-dimensional reorientation. This system reveals general principles of how molecular machines balance speed, torque, and switching in complex environments.

## INTRODUCTION

**Fig 1:**
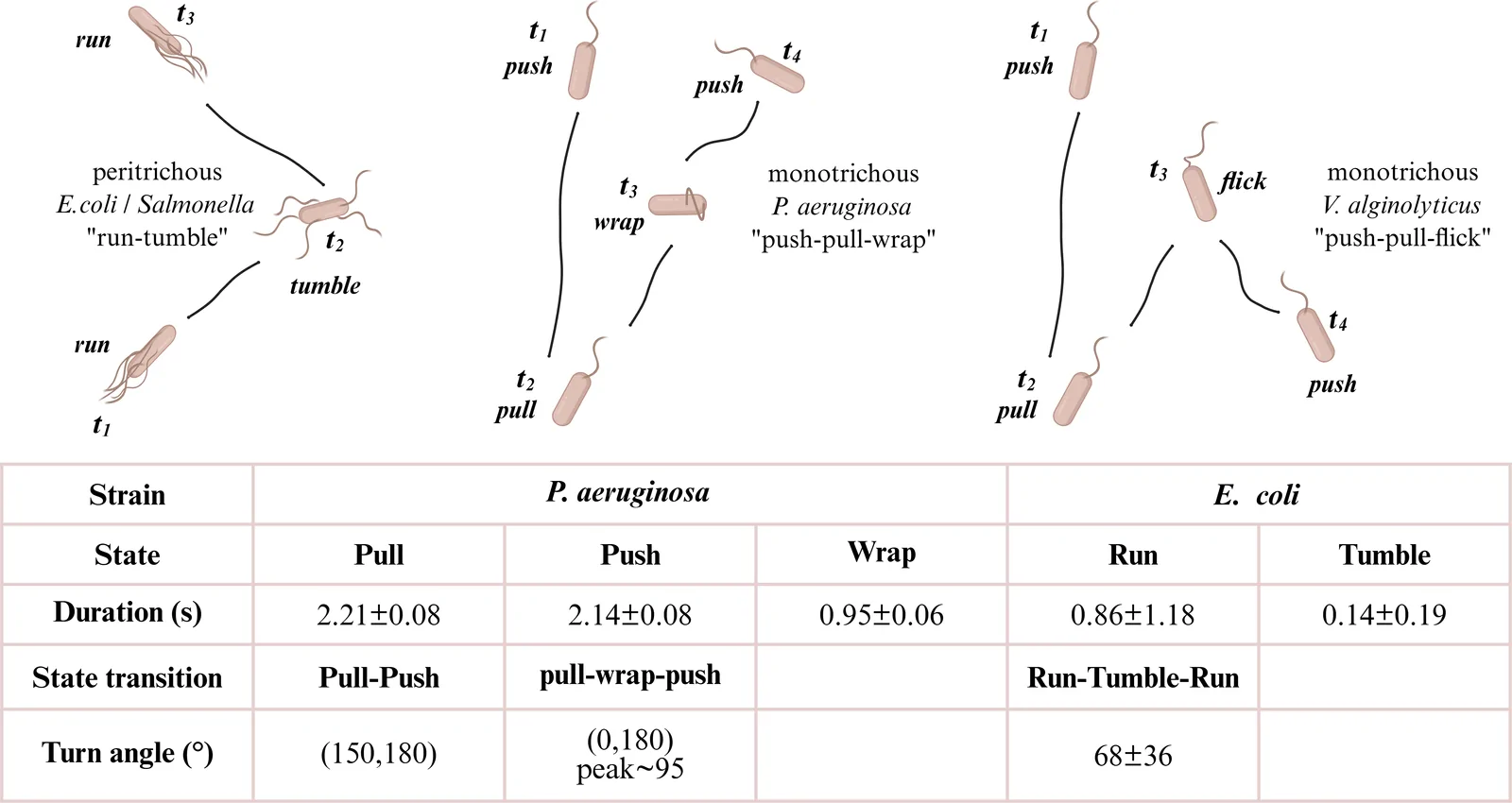
Distinct motility strategies of representative flagellated bacteria (from left to right: *E. coli*/*Salmonella*, *P. aeruginosa*, and *V. alginolyticus*). The associated kinetic parameter data are compiled from published literature (7, 8).

The bacterial flagellar motor is one of the most sophisticated nanomachines in nature (1, 2). *Escherichia coli* is often used as a model organism for studying flagellar motors, which achieve chemotaxis by adjusting the motor’s clockwise (CW) bias to alter the cell’s swimming state (3). This bias is set by the phosphorylated CheY (CheY-P), which binds the switch protein FliM on the cytoplasmic C-ring and thereby promotes CW rotation (4); the level of CheY-P is in turn continuously adjusted by the upstream chemotaxis signaling system as the cell samples chemoeffector gradients, allowing the cell to bias its run/tumble transitions toward favorable directions. While this paradigm is highly effective for peritrichously flagellated bacteria with biased motors, it does not fully explain the locomotion strategies of monotrichously flagellated bacteria—a group in which different species have evolved markedly distinct mechanical solutions. For instance, *Vibrio alginolyticus* employs a “run–reverse–flick” mechanism that relies on a buckling-driven flagellar flick to randomize swimming direction (5), whereas *Pseudomonas aeruginosa* has adopted a fundamentally different strategy based on symmetric modulation of rotational dwell times (6). These contrasting strategies are illustrated in Fig. 1. This mechanistic distinction from other swimmers makes *P. aeruginosa* an ideal model for studying how flagellar structure, stator remodeling, and signaling networks integrate to achieve efficient directional migration while the motor remains near unbiased.

Furthermore, the flagellar motor of *P. aeruginosa* is mechanistically rich, making it an attractive biophysical model. This motor is powered by two sets of proton-driven stator systems: MotAB and MotCD. Although both stator systems are independently capable of driving flagellar rotation, they are functionally non-identical, with distinct mechanical properties (torque, speed stability, and load-dependent behavior) that are discussed in detail below (9–12). Recent studies have demonstrated that these stators differ in their load-dependent torque contributions and rotational speed stability. MotAB provides higher torque under low-load conditions, whereas MotCD outperforms MotAB near stall, consistent with their distinct torque–speed relationships. Moreover, the wild-type motor remodels its stator composition in response to the surrounding mechanical environment (11, 12). Structural biology studies have provided additional insight, revealing that the motor of *P. aeruginosa* possesses a suite of structural features associated with its dual proton-driven stator configuration—including the refined architecture of the periplasmic ring and the presence of specific assembly intermediates—features that are thought to stabilize the motor’s high-torque operational state (13–15).

Many revealing studies on this system have employed methods that connect single-motor output to whole-cell motion. Techniques such as bead assays, tethering assays, optical trapping, microfluidics, holographic 3D tracking, and near-surface imaging have made it possible to measure torque generation, switching characteristics, stator-specific performance, and swimming trajectories in the same biological system (6, 11, 12, 16–19). Together, these studies support a coherent picture: the flagellar motor of *P. aeruginosa* is not merely a propulsion device but a tunable sensor–actuator. Its near-unbiased switching, dual-stator mechanics, and surface-responsive signaling help the cell navigate through diverse environments, with the motor itself increasingly recognized as a mechanosensor that feeds back onto cellular decision-making (6, 16, 20–22).

## *P. AERUGINOSA* AS A UNIQUE MOTOR MODEL

In canonical peritrichous systems, chemotaxis is traditionally described as the modulation of motor CW bias to alter run length and tumble frequency in response to stimuli. That logic does not transfer cleanly to a monotrichous swimmer. A singly flagellated cell cannot execute a true run-and-tumble trajectory, and a strong directional bias in such a cell would cause a randomly oriented population to respond incorrectly to an external gradient.

By combining cell-tethering assays with microfluidic chemotaxis measurements, researchers discovered that *P. aeruginosa* instead employs a distinctly different chemotactic strategy: while maintaining an essentially constant CW bias of ~0.5, it symmetrically modulates the dwell times of the CW and counterclockwise (CCW) states in response to chemoeffector gradients (6). Thus, the value of this system lies not only in its experimental tractability but also in its conceptual implications—defining a new chemotactic response paradigm at the single-motor level. Notably, this strategy is also distinct from that of another well-studied monotrichous swimmer, *Vibrio alginolyticus*, which adopts a run–reverse–flick mechanism in which a buckling-driven flick of the flagellum following the reverse step randomizes the next swimming direction (5, 23). The distinction extends beyond this flick-mediated reorientation. Unlike the near-unbiased motor of *P. aeruginosa*, the wild-type *V. alginolyticus* motor is intrinsically CCW biased, rotating with a CW:CCW ratio of approximately 1:3 (24). Rather than maintaining a CW bias near 0.5 through symmetric modulation of CW and CCW dwell times, *V. alginolyticus* regulates chemotaxis by modulating motor switching rates to prolong swimming intervals in favorable directions. In addition, *V. alginolyticus* employs additional regulatory components such as ZomB, which is essential for CW rotation and functions alongside CheY in rotational control (25). Together, these contrasting solutions highlight that monotrichous bacteria have evolved fundamentally different strategies, at both the behavioral and the motor-regulatory levels, to circumvent the limitations imposed by a single polar flagellum. Critically, near-unbiased switching appears not to be a universal feature of monotrichous bacteria, but rather a solution specific to *P. aeruginosa*, which represents a conceptually parsimonious example of switching-kinetics-based chemotactic control: because CW bias remains fixed near 0.5, only a single variable—the overall switching rate—needs to be modulated to symmetrically adjust dwell times, whereas *E. coli*-type systems require coordinated changes in both CW bias and switching rate.

This distinct control logic is implemented by an equally distinct physical machine. *Pseudomonas* species carry a single polar flagellum rather than the peritrichous arrangement found in enteric model systems. This strict spatial and numerical control is governed by specific regulators such as FlhF (which determines polar localization) and FleN (which restricts flagellar number to one) (26, 27), integrated within a four-tiered transcriptional program instead of the simpler cascade familiar from enteric bacteria (28, 29). In *P. aeruginosa*, the motor is further specialized by the presence of two proton-driven stator systems and by structural elaborations characteristic of high-load polar motors (10, 30). These features, together with the availability of single-cell assays for measuring kinetic output, stator occupancy, and swimming behavior, establish this organism as a highly tractable model system for integrating molecular motor physics with cellular behavior (11, 12, 14).

## DUAL-STATOR ARCHITECTURE AND STRUCTURAL SPECIALIZATIONS

The *P. aeruginosa* flagellum retains the core three-part organization shared by many gram-negative bacteria: a basal body embedded in the cell envelope, a hook that serves as a flexible universal joint, and a helical filament that acts as the propeller (28). What makes the system especially notable is not the basic parts list but the way torque is generated and stabilized.

### Dual proton-driven stator system

Genetic and functional studies have established that the motor is driven by two distinct H^+^-driven stator sets, MotAB and MotCD (9, 10). Early analyses showed that deletion of either stator pair reduced motility in different ways, while their combined loss abolished swimming, supporting the view that the two stator systems are partly redundant yet physiologically distinct (10). That observation has matured into a more nuanced picture in which the motor acts as a mixed-stator machine, with composition remodeled in response to load and environmental changes (11, 12). This view builds on foundational observations in *E. coli*, where individual stator units were shown to undergo continuous stochastic turnover at active motors and to assemble in a load-dependent manner (31–33), thereby establishing stator dynamics as a general organizing principle of bacterial flagellar motors. *P. aeruginosa* appears to exploit this principle with added complexity, as it harbors two compositionally distinct stator species whose relative occupancies can be tuned by mechanical context.

Single-motor bead assays indicate that MotAB and MotCD differ substantially in their torque-generating properties: motors driven solely by MotAB produce higher overall torque—though whether this reflects greater torque generation per stator unit, higher stator occupancy, or both remains unknown—whereas motors driven solely by MotCD exhibit more stable speed output (i.e., lower speed variability), the mechanistic basis of which remains to be elucidated (12). Optical trap-based torque–speed measurements deepen this distinction by showing different load dependencies for the two stator systems. In these measurements, MotAB displayed slip-bond behavior, whereas MotCD showed catch-bond behavior (11). This catch-bond mechanism—initially discovered in the mechanosensitive motors of *E. coli*—allows the stator to anchor more tightly to the peptidoglycan under high load (34, 35). The wild-type motor behaved as though it could combine contributions from both stator types, with total torque close to the sum of the two single-stator systems (11). The simplest interpretation is that stator diversity expands the mechanical operating range of the motor. Instead of being genetically restricted to either a fast, low-load or a high-load mode, *P. aeruginosa* can mix stator species within a single motor and shift their balance in response to changing conditions.

### Motor structure refinement adapted to the dual-stator system

Cryo-electron tomography placed these physiological findings within a structural context. Studies have revealed that motors with dual H^+^-driven stators (including those of *P. aeruginosa*) exhibit smaller elaborations of their P-rings beyond the core L- and P-rings of simpler H^+^-driven motor systems (30). This correlation suggests that stator type and periplasmic scaffold structure coevolved. Therefore, structural diversity is not merely decorative; it may contribute to the motor’s efficient recruitment, stabilization, or load transfer under a wider range of mechanical conditions.

Additional *in situ* tomography of *P. aeruginosa* polar flagella has revealed species-specific features of polar motors and captured incomplete subassemblies that illuminate assembly and disassembly processes. Broader *in situ* studies across related motors have revealed stable outer membrane relics after disassembly and transient cytoplasmic ring structures during assembly, with the latter appearing necessary for proper stator ring formation in high-torque motors (13, 15). Together, these studies reinforce an important theme: the dual-stator motor of *P. aeruginosa* is a dynamically built and rebuilt machine whose structural accessories are tightly coupled to torque generation.

Complementing these *in situ* tomographic findings, near-atomic resolution single-particle cryo-EM reconstructions of isolated stator complexes from related H^+^-driven motors have revealed an unexpected 5:2 subunit stoichiometry, in which a pentameric MotA ring rotates around a dimeric MotB shaft to drive C-ring rotation through a “gear-like” mechanism (36, 37). This structural framework provides a molecular basis for interpreting the distinct mechanical phenotypes of MotAB and MotCD measured by optical trapping (11), and it raises important open questions about how these two structurally homologous but non-identical stator species coexist and share mechanical load within a single-motor scaffold—specifically, how their relative occupancy is regulated to achieve optimal motor performance under varying conditions.

### Methods for probing motor output across scales

**Fig 2:**
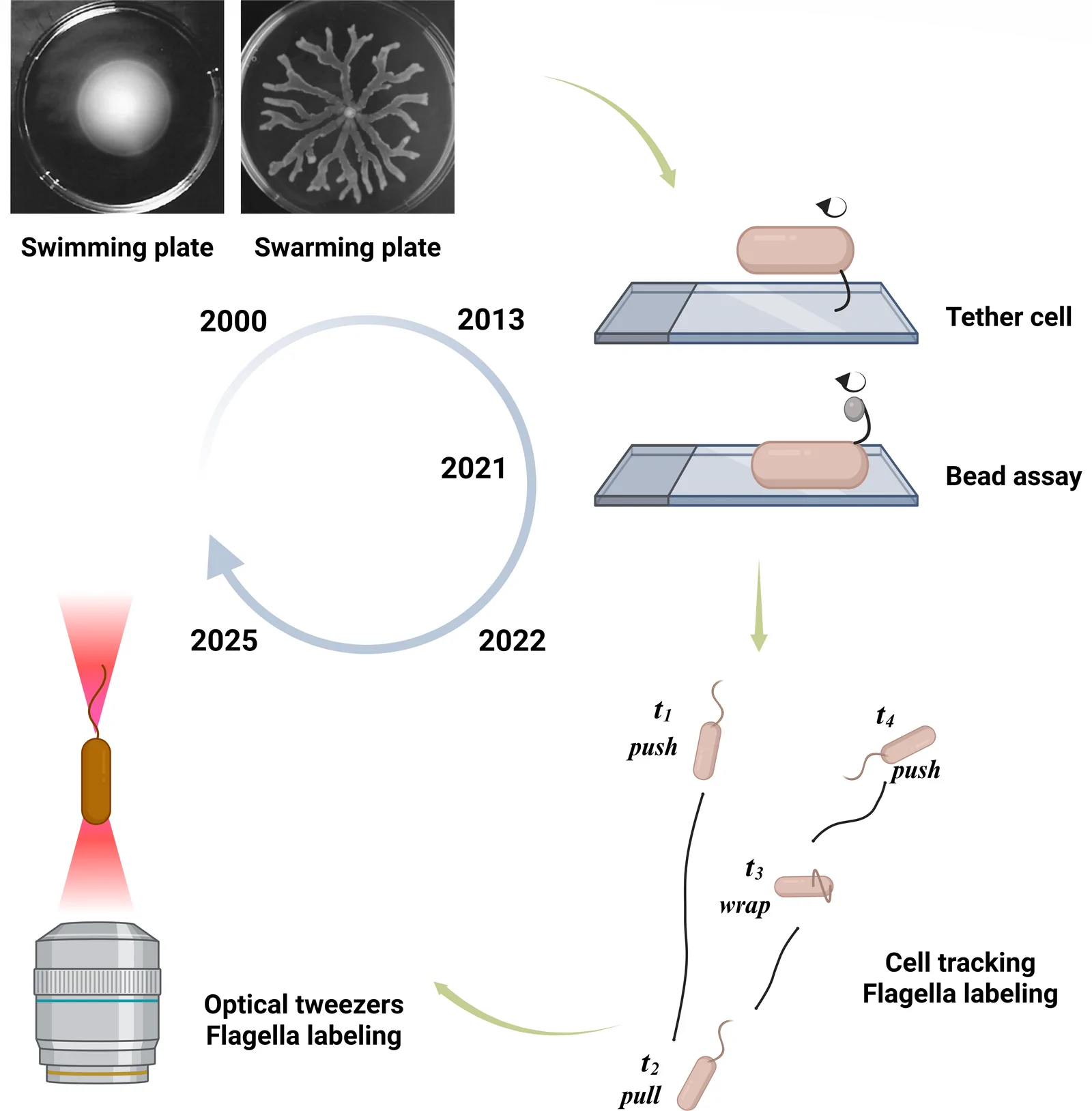
The evolution of research methods for studying the output dynamics of the flagellar motor and the swimming behavior of *Pseudomonas aeruginosa*, showing a temporal progression from macroscopic to microscopic scales and from laboratory settings to quasi-natural environments. The images of swimming and swarm plates were sourced from previously published literature (38, 39).

A defining strength of the contemporary *P. aeruginosa* motility field is that its experimental toolkit now spans the full range from molecular motor biophysics to whole-cell behavior. Since no single detection method can fully capture the complexity of an entire system, the most insightful studies often emerge from the integrated application of multiple approaches (Fig. 2).

### From macroscopic motion characterization to single-cell tracking

The classical approach to assessing cell motility is the swimming-plate assay, which is readily applicable to *P. aeruginosa*. By comparing the diameters of chemotactic rings formed on the surface of soft-agar nutrient plates, one can perform a quasi-quantitative comparison of motility between wild-type and mutant strains (9, 10, 40). Swarming assays, conducted on plates with higher agar concentrations, provide a complementary readout that indirectly reports on the swimming efficiency of mutants under high-load conditions (10, 38, 40). These plate-based assays remain indispensable for rapid phenotypic screening, but they operate on population averages and therefore cannot resolve behavior at the single-cell level.

Single-cell tracking complements these population-level assays by enabling behavioral analysis at the level of individual cells. Observing individual bacteria swimming in a bulk liquid environment allows for the most faithful reconstruction of their native swimming modes, and many intriguing claims regarding unbiased motor operation have originated from such observations. Conventional phase-contrast microscopy enables preliminary characterization of basic motility parameters, such as swimming speed, at the single-cell level (9, 10). Holographic three-dimensional tracking extends this capability by resolving multiple swimming patterns—including smooth (relatively straight trajectories during prolonged runs), oscillatory (side-to-side wobbling, likely reflecting frequent motor reversals), and helical (corkscrew-like trajectories arising from the interplay between cell-body rotation and flagellar propulsion) states—and capturing the transitions between them (19). These trajectory-level patterns are distinct from the flagellar “wrap” state, in which the filament coils around the cell body during push-to-pull transitions, generating large reorientation angles. Near-surface imaging methods, such as differential interference contrast microscopy, total internal reflection fluorescence microscopy, and multimode microscopy, have revealed how stator composition and surface proximity affect bacterial speed, directionality, and attachment behavior (16, 17, 41). More recently, fluorescent labeling of the flagellar filament was achieved via maleimide–dye conjugation to cysteine residues introduced into FliC, allowing visualization of flagellar dynamics in real time (7). Moreover, this approach has further facilitated single-cell tracking within crowded, swarm-like environments (42). Nevertheless, none of these approaches can directly report the instantaneous rotational state of the motor, and practical limitations such as defocusing continue to make long-term, stable monitoring of motor output difficult.

### Powerful tools for single-motor monitoring: cell-tethering and bead assays

More than a decade ago, the Chiam group successfully implemented cell-tethering assays in *P. aeruginosa* and developed a dedicated trajectory analysis algorithm to characterize motor output with improved accuracy. Their work revealed that deletion of the central chemotaxis response regulator CheY markedly reduced the frequency of motor switching, thereby establishing a regulatory link between the chemotaxis signaling network and the flagellar motor (18). Subsequently, Cai et al. combined this method with microfluidics to perform chemotaxis response experiments at the single-cell level, demonstrating that *P. aeruginosa* achieves chemotaxis by symmetrically modulating the dwell times in both rotational directions (6). The polar arrangement of the flagellum, however, causes tethered cells to rub against the interface during rotation and destabilizes the cell’s center of rotation, compromising long recordings. A method capable of stably monitoring flagellar motor output over extended periods was therefore needed.

That capability arrived with the adaptation of the bead assay to *P. aeruginosa*, in which flagellar filament stubs are labeled with microbeads via biotin–streptavidin coupling (12); this approach has since become the definitive means of quantifying single-motor parameters in this organism. In brief, streptavidin-coated magnetic microbeads are attached to the biotinylated stub of a truncated flagellar filament, and the rotation of the bead is used to infer motor operation under a defined viscous load. This assay makes it possible to compare wild-type and flagellum-related mutant strains within the same mechanical framework and to quantify differences in torque generation, speed stability, and switching dynamics (12, 43, 44). Its value in *P. aeruginosa* is twofold. On the one hand, it overcomes limitations inherent to macroscopic motility measurements, which often conflate genuine motor phenotypes with confounding factors such as growth rate, surface humidity, and collective behavior. On the other hand, it resolves difficulties encountered by other single-motor techniques, including load instability and limited observational continuity.

At the same time, bead-based observation is best understood as a controlled perturbation rather than a direct reflection of native motor function. Bead size sets the viscous load; filament truncation alters the geometry of the propulsion unit; and surface immobilization removes the long-range hydrodynamic environment experienced by free-swimming cells. For these reasons, the most informative recent studies have combined single-motor detection with free-swimming or near-surface measurements, leveraging the complementary strengths of each approach to interpret motor behavior in a more physiologically meaningful context (12, 16).

### Torque–speed measurement by optical trapping

Ideally, motor research should be performed under conditions that preserve the physiological state of the cell as closely as possible while still permitting rigorous quantification. Optical trapping meets this dual requirement particularly well, allowing the motor to exhibit mechanical phenotypes that more faithfully reflect its native operating regime. In a recent study, optical tweezers were combined with fluorescent labeling of the flagellar filament to map the torque–speed curves of wild-type *P. aeruginosa* and single-stator mutants (strains lacking either MotAB or MotCD, thus containing only the remaining stator type). Load was varied by adding different concentrations of Ficoll to the motility buffer to alter its viscosity. The torque–speed curves of MotAB and MotCD revealed clear differences: MotAB exhibited a low stall torque with a sharp rise to peak torque, a broad plateau, and an exceptionally high zero-load speed, whereas MotCD displayed a high stall torque with a gradual decline followed by a steep final drop, resulting in a narrower working range. Because the torque–speed relationship is one of the most fundamental characteristics of the flagellar motor and largely dictates its mechanical output, this represents a significant methodological advance. Applied to *P. aeruginosa*, this approach further revealed differences in the mechanistic roles of MotAB and MotCD, particularly their distinct phenotypic responses to load changes (11). The importance of this method lies in its ability to transform our understanding of the dual-stator system from qualitative genetic observations into quantitative biophysical insight. Its principal drawbacks are that it is highly instrument-dependent and demands considerable experimental expertise, which together limit its widespread adoption.

## REGULATION OF AN UNBIASED MOTOR

**Fig 3:**
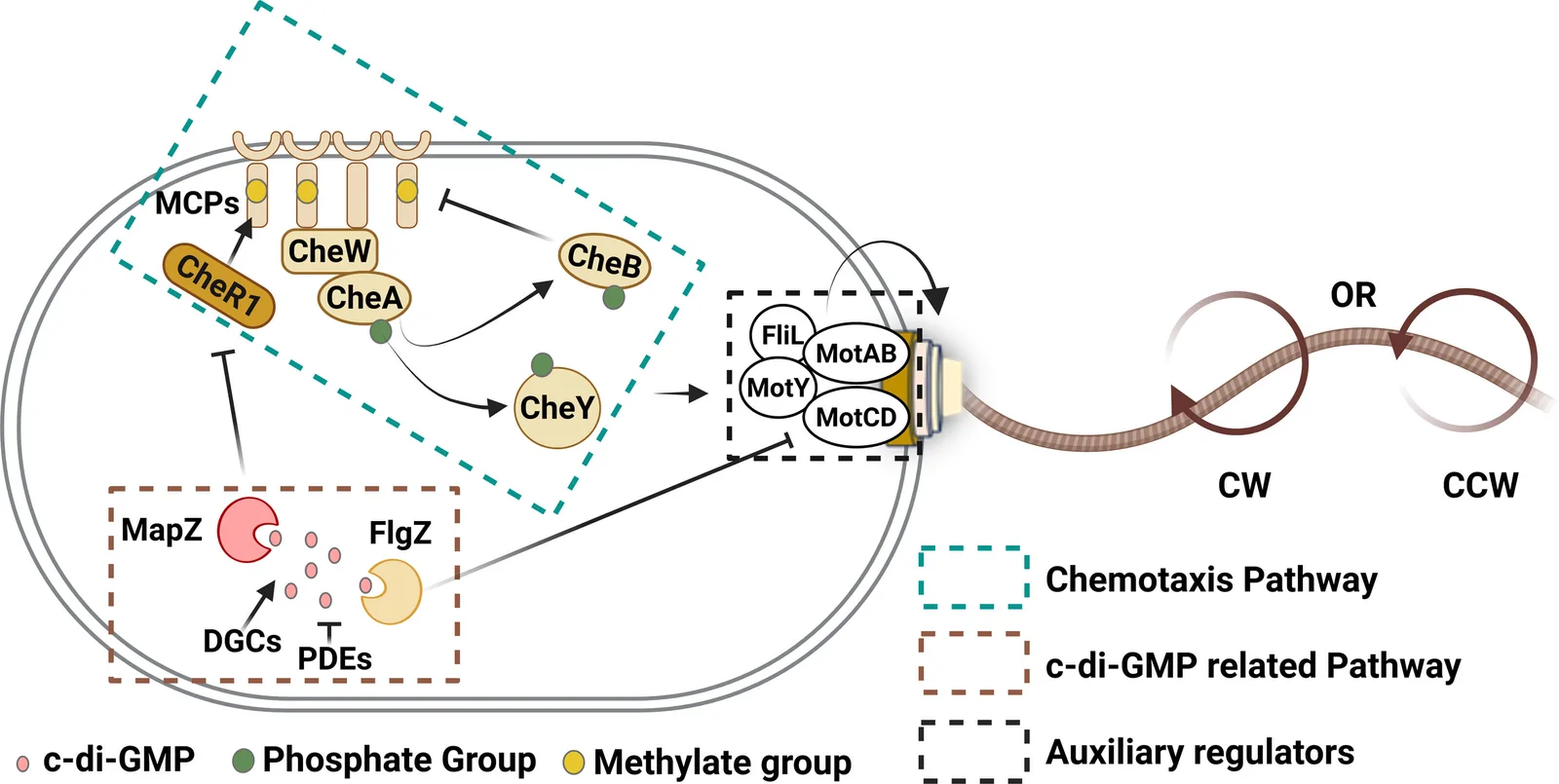
The main pathways currently known to regulate the unbiased motor operation of *P. aeruginosa*.

In bacteria with a variable-bias motor, such as *E. coli*, rotational bias is not fixed but is dynamically modulated by the intracellular chemotaxis signaling network. Specifically, CheY-P binds to the C-ring motor proteins FliM and FliN, inducing a conformational change that promotes CW rotation. The intracellular pool of CheY-P is, in turn, tightly controlled by the upstream chemoreceptor complex (MCP-CheA-CheW) and the adaptation system (CheR and CheB) (3). *P. aeruginosa* possesses a considerably more complex signal transduction network (45), and its motor displays unbiased rotational behavior. This raises an obvious question: what factors govern the output of such a motor? Figure 3 summarizes the currently recognized pathways that regulate motor switching kinetics and stator composition in *P. aeruginosa*, and the underlying mechanisms are discussed in detail below.

### Symmetric regulation of dwell times by the chemotaxis system

The clearest answer to date is that, under the control of its chemotaxis system, *P. aeruginosa* regulates its CW and CCW dwell times symmetrically. When cells in a chemoattractant gradient move up-gradient, both dwell times increase, extending the duration of smooth-swimming runs and thereby enhancing net up-gradient progression; when they move down-gradient, both dwell times decrease, leading to more frequent reversals and more effective reorientation; yet the CW bias remains essentially constant at approximately 0.5 (6). In a *cheY* knockout strain, motor switching frequency is markedly reduced, strongly indicating that this process is indeed under chemotaxis control, albeit through a pathway distinctly different from that operating in biased motors (18).

For a monotrichous bacterium carrying a single polar flagellum, this represents an elegant solution. It allows the cell to alternate between forward and backward propulsion while preventing the population from becoming locked into a biased orientation distribution. This finding reframes chemotactic regulation in *P. aeruginosa*: rather than modulating both mean CW bias and switching rate together, as in *E. coli*, chemotaxis here is achieved solely through the dynamic adjustment of motor switching rates while the mean bias remains fixed—a distinction that reflects the fundamentally different control logic employed by unbiased motors. The key variables thus become how long the motor persists in each rotational state and how the mechanical context feeds back onto these timescales.

### c-di-GMP as a regulator of motor output

It is well established that the c-di-GMP signaling network and the chemotaxis system are intimately intertwined, collectively determining bacterial motility patterns and behavioral decisions, particularly in the trade-off between planktonic swimming and surface colonization. Recent studies have demonstrated that c-di-GMP signaling acts on the flagellar motor at two distinct levels: switching and speed. Elevated c-di-GMP levels inhibit motor switching and reduce rotational speed, thereby lowering both reversal frequency and swimming velocity. This process depends on binding of c-di-GMP to the adaptor protein MapZ, which in turn inhibits the methyltransferase CheR1, and is itself subject to regulation by chemotaxis pathways (46), suggesting that motor switching kinetics are embedded within a broader signaling network rather than being governed by a single phosphorelay system. c-di-GMP can also bind to the PilZ-domain protein FlgZ, which interacts directly with the MotCD stator to reduce motor speed and torque, thereby slowing bacterial motility in response to elevated c-di-GMP levels (47). The concept that local pools of c-di-GMP regulate motor switching is particularly compelling for monotrichous bacteria, as it provides a mechanism for rapid, spatially confined modulation of motor output without requiring global changes in cytoplasmic status (48). Previous work has revealed extensive spatial colocalization between chemoreceptor complexes and the flagellar motor, and additional evidence indicates that CheY can influence c-di-GMP production through a pathway that intersects canonical chemotaxis signaling. Accordingly, we propose that the chemotaxis network orchestrates local and precise regulation of motor output (49).

Work on surface adaptation extends this logic. Surface attachment triggers a rapid pathway involving FlhF, FimV, and cAMP signaling that leads to cessation of flagellar rotation soon after contact (22). Other studies show that environmental ethanol inhibits swimming and swarming through MotAB, PilZ-domain proteins, and diguanylate cyclases, including SadC and GcbA, thereby linking external chemical context to stator-linked c-di-GMP control (50). Meanwhile, stator components that have disengaged from the flagellar motor can stimulate c-di-GMP synthesis through interactions with SadC, creating a positive feedback loop that stabilizes reduced motility and biofilm-associated behavior (51). Taken together, these findings place the *Pseudomonas* flagellar motor within a larger decision-making system: the motor not only is regulated by second messengers but also contributes to generating the signals that determine whether motility should persist.

### Auxiliary regulators of motor output

Existing research indicates that the stator system does not dictate the dynamic output of the *P. aeruginosa* flagellar motor in isolation. Recent work has confirmed that auxiliary proteins, specifically FliL and MotY, play important regulatory roles. FliL is generally considered to assist motors with only a single stator type (i.e., MotAB-only or MotCD-only motors) in coping with high-load environments (52). However, bead assays reveal that FliL exerts distinct effects on the two stator systems of *P. aeruginosa*. It promotes motor torque generation and maintains the stability of speed output through MotCD while simultaneously helping MotAB maintain a high switching frequency (44).

MotY is a component associated with the periplasmic ring structure of monotrichous bacteria and has likewise been implicated in shaping motor output (53). Studies in *P. aeruginosa* have shown that MotY improves stator integrity and assembly efficiency, maintains torque, and regulates motor switching frequency. Notably, it can induce a counterclockwise rotational preference in otherwise unbiased motors (43). This observation is informative because it shows that motor behavior is not dictated by CheY-P alone but emerges from the combined action of switching-control factors, stator composition, and auxiliary proteins that govern stator engagement and mechanical transmission.

## UNBIASED MOTOR-DRIVEN SWIMMING BEHAVIOR

Cell swimming is the direct phenotypic manifestation of flagellar motor operation. Owing to the polar, monotrichous flagellar arrangement of *P. aeruginosa*, the run-and-tumble framework developed for peritrichous bacteria cannot explain its movement. Recent technological advances have gradually elucidated its swimming strategies for achieving efficient chemotaxis, as well as its motile behavior in specialized environments such as near surfaces.

### Swimming strategy in bulk liquid

As introduced above, *P. aeruginosa* swimming is characterized by a “run–reverse” pattern in which forward (push) and backward (pull) runs are separated by motor reversals. In this section, we focus on recent mechanistic insights into this swimming behavior, particularly the discovery of the flagellar wrap state. Early single-cell tracking and cell-tethering assays also proposed a “pause” state in addition to run and reverse (6, 18). However, owing to a long-standing lack of direct observational evidence, there has been considerable controversy regarding whether the pause mode truly exists and what functional role it might play. Using the bead-labeling experiment described above, we found that the motor can actually rotate continuously for tens of seconds, suggesting that the previously reported pause mode likely originates from unintended physical contact between the cell body and the surface.

By combining gene editing and live-cell fluorescent labeling of the flagellar filament, we achieved real-time simultaneous observation of single-cell trajectories and dynamic flagellar behavior (7). Surprisingly, we discovered a distinct swimming mode—a wrap state—in which the flagellar filament wraps around the cell body and the cell swimming speed is markedly reduced. This state parallels coiling/wrapping behaviors previously documented in other polarly flagellated species, including *Helicobacter suis*, *Pseudomonas putida*, and *Shewanella putrefaciens* (54–57). In *P. aeruginosa*, the wrap state occurs during the transition from the “pull” state to the “push” state, and its onset is attributed to a buckling mechanical instability of the hook. Cells in the wrap state exhibit a larger cell-body turning angle compared to cells in the push/pull state or those undergoing Brownian motion, with a broad turning-angle distribution peaking near 90°, which facilitates reorientation in three-dimensional space and enables efficient chemotaxis (7). Subsequent chemotaxis simulations have further corroborated this finding (7).

From the perspective of motor control, near-unbiased switching is eminently sensible in this context, particularly because *P. aeruginosa* relies on large-angle reorientation events, such as the wrap state described above. A cell that uses reversals and such large-angle reorientation events gains little from strongly favoring one rotational state; what it requires instead is a means of modulating persistence length without sacrificing directional flexibility. Symmetric adjustment of CW and CCW dwell times provides exactly this capacity, thereby unifying the internal regulatory logic of the flagellar motor with the external dynamic phenotype of the flagellar filament.

It is worth noting that a particularly interesting niche exists in the transition from bulk liquid to biofilm formation: cell movement near the interface. *P. aeruginosa* typically first contacts a surface using its polar flagellum and then either detaches or initiates its surface-attachment program. This near-surface behavior also helps explain why *P. aeruginosa* serves as an excellent model for unbiased motor dynamics. A motility-switching system that remains close to unbiased is well suited for the repeated cycles of approach, retreat, and reorientation that occur near interfaces, where excessive directional preference would incur unnecessary energetic costs. In this way, the same system that supports efficient movement in bulk liquid also supports surface sampling and early biofilm formation.

### Motility on semisolid surfaces

Unlike free swimming in bulk liquid—where cells experience minimal mechanical resistance—movement on semisolid surfaces represents a specialized form of bacterial locomotion within confined, viscous, or gel-like matrices. Importantly, this mode of motility is not merely a laboratory construct; it mirrors conditions bacteria encounter in natural habitats such as hydrated soil interfaces, mucosal surfaces of host tissues, biofilm interstitial spaces, and decaying organic matter, where bacteria frequently navigate environments that are neither fully liquid nor solid. In swimming plates (typically containing 0.3% agar), bacteria move through a low-viscosity semisolid matrix as individuals, forming a uniform, radially expanding circular zone. The diameter of this zone serves as a quantitative readout for motor output and chemotactic efficiency (9, 10). In contrast, swarm plates (containing 0.5%–0.7% agar with rich nutrients) support a distinct mode of surface motility: cells swim collectively within a thin fluid layer on top of the agar surface, rather than through the agar matrix itself. This fluid layer is maintained by osmotic agents and surfactants secreted by the cells (58). To overcome surface tension, the flagellar systems of some bacteria undergo differentiation and secrete biosurfactants such as rhamnolipids, enabling coordinated “rafting” migration, which manifests macroscopically as complex, dendritic patterns (38, 59). It is worth noting, however, that *P. aeruginosa* itself does not undergo such differentiation to produce numerous additional polar flagella or lateral flagella, although as many as two polar flagella have been observed under conditions of mechanical stress. Instead, it appears to rely primarily on dynamic stator remodeling, reflected by an increased proportion of MotCD stators, as a key mechanism for sustaining motility in environments of elevated viscosity (9, 10).

## OUTLOOK

*P. aeruginosa* has become more than an alternative to the canonical enterobacterial flagellar systems; it has emerged as a distinct and revealing model for studying how a bacterial motor can remain near unbiased while still supporting efficient chemotaxis, rich trajectory switching, and rapid adaptation to surfaces. The key to this behavior lies in the combination of motor architecture and control logic: a single polar motor built around two proton-driven stator systems, structurally reinforced by specialized ring elements, and regulated through coupled switching, stator remodeling, and nucleotide signaling.

The field has advanced rapidly because methods now span the full scale of the problem. *In situ* structural imaging defines the machine; bead assays and optical trapping measure its mechanics; and tethering, microfluidics, and single-cell tracking reveal how those mechanics are deployed during navigation. Together, these approaches support a clear conclusion: the flagellar motor of *P. aeruginosa* is a valuable model system not despite its departure from the textbook bias-control paradigm but because of it. Its near-unbiased motor demonstrates that efficient bacterial navigation can be achieved through symmetric control of switching kinetics embedded within a mechanically adaptive dual-stator motor.

Nevertheless, many questions regarding this motor remain unresolved, and several lines of inquiry appear particularly pressing.

### Quantitative delineation of the switching mechanism of unbiased motors

In previously constructed *cheA* and *cheY* mutant strains, the flagellar motor switching frequency is significantly reduced, strongly suggesting that CheY-P participates in regulating unbiased motor switching and directly modulates the switching rate. However, because intracellular CheY-P levels are difficult to manipulate independently, the absolute quantitative relationship between CheY-P concentration and motor switching frequency remains elusive. Drawing on established strategies that ectopically express a phosphomimetic CheY variant (e.g., CheY^**^) (60), it may become possible to delineate this absolute relationship. This would provide an essential experimental foundation for constructing theoretical models of motor dynamics in this class of systems.

### Simultaneous measurement of structural and dynamical properties at the single-motor level

With advances in cryo-electron microscopy, a series of distinctive structural features of the *P. aeruginosa* flagellar motor—including the dual-stator architecture and a range of assembly intermediates—have been revealed. In parallel, bead assays, cell tethering, and optical tweezers have enabled the monitoring of single-motor dynamic outputs and the quantification of mechanical properties. What is still missing, however, is a direct mapping from instantaneous stator occupancy and accessory-ring state to torque generation and switching statistics in living cells; conclusions drawn purely from ensemble statistics remain insufficiently direct.

### Motor operation and motor-driven motility in realistic environments

The unbiased motor powered by the *P. aeruginosa* dual-stator system appears to adapt dynamically to changes in mechanical load arising from diverse physical sources, including viscosity, flagellar geometry, surface proximity, and physical confinement. Notably, these mechanical cues appear to be sensed and integrated into downstream regulatory circuits rather than acting on the motor in isolation: for instance, pore confinement triggers a distinct pause motility mode characterized by repeated reorientation events that facilitate escape, a behavior mediated by elevated intracellular c-di-GMP levels and dependent on the PilZ-domain effector FlgZ (61). There is an urgent need to extend motor measurements into more host-like and polymicrobial conditions to clarify why this architecture and operational paradigm are favored in infection-relevant habitats. Furthermore, some clinical isolates exhibit external phenotypes distinct from those of laboratory model strains. For instance, certain isolates from patients with chronic respiratory infections such as cystic fibrosis show overproduction of polysaccharide polymers, resulting in a mucoid colony morphology (62). The actual motor and motility characteristics of such strains remain largely uncharacterized and represent a promising avenue of study.

Addressing these questions will not only deepen our understanding of *P. aeruginosa* pathophysiology but also yield general principles of molecular machine design. The unbiased flagellar motor represents a convergent solution to the problem of navigating complex, structured environments—one that differs fundamentally from the canonical bias-based control of peritrichous bacteria. By integrating precise single-motor biophysics with *in vivo* structural dynamics and ecologically relevant behavioral assays, the field is poised to uncover how mechanical forces, regulatory networks, and structural adaptations coalesce to produce robust and versatile motility. Ultimately, *P. aeruginosa* offers a compelling paradigm: even a seemingly “simple” bacterial motor can embody sophisticated engineering trade-offs, balancing speed, torque, switching, and unbiased operation to thrive in hostile and changing habitats.
